# Analytical Performances of Polymer-Based Biosensors for Real Samples Application

**DOI:** 10.3390/bios16040207

**Published:** 2026-04-05

**Authors:** Marcello Mascini, Sara Palmieri, Fabiola Eugelio, Maikel Izquierdo Rivero, Michele Del Carlo

**Affiliations:** Department of Bioscience and Technology for Food, Agriculture and Environment, University of Teramo, 64100 Teramo, Italy; spalmieri@unite.it (S.P.); feugelio@unite.it (F.E.); mizquierdorivero@unite.it (M.I.R.); mdelcarlo@unite.it (M.D.C.)

**Keywords:** polymer biosensors, conductive polymers, molecularly imprinted polymers, hydrogels, matrix effect, optical and electrochemical detection, real samples

## Abstract

Polymer-based biosensors have evolved from passive supports into active functional elements that dictate analytical performance in complex real-world samples. This critical review with meta-trend analysis examines 96 original research articles published between 2015 and 2025, evaluating how four polymer classes (conductive polymers, redox-mediator polymers, hydrogels, and molecularly imprinted polymers) address matrix effects in food, beverage, environmental and clinical applications. Electrochemical detection dominates (79% of studies), with conductive polymers enabling low-potential operation that excludes electroactive interference. Hydrogels achieve superior precision (RSD below 3%) in protein-rich matrices through biocompatible microenvironments that preserve enzyme kinetics. Molecularly imprinted polymers provide unmatched stability in harsh environments for trace-level detection of heavy metals and toxins, though delayed response times from slow analyte diffusion persist. Critical evaluation exposes validation deficits: 91% of studies omit limits of quantification, while approximately one-third lack reproducibility (33%) and precision (30%). The multi-matrix challenge, maintaining calibration across different hostile environments, remains the primary barrier to commercial deployment. Advanced architectures, including nanocapsulation, hierarchical nanocomposites, and microneedle-integrated systems, offer pathways to overcome limitations in fouling resistance and operational stability.

## 1. Introduction

Polymer-based biosensors have become essential tools in modern analytical chemistry. Recent literature has established their ability to navigate the chemical complexities of food, environmental, and clinical matrices [1]. Previous comprehensive reviews have analyzed electroactive analyte detection [2], screening strategies for malignancies [3,4,5], and requirements for high accuracy in cardiovascular and metabolic monitoring [6,7,8].

Managing complex matrices represents a significant focus in existing literature. Reviews on anti-fouling polymers [9] and conducting polymer composites [10,11,12] highlight strategies for maintaining analytical integrity in blood, urine, and saliva. Environmental monitoring [13,14] and food safety applications [15] have demonstrated that molecularly imprinted polymers (MIPs) provide essential stability and selectivity. This framework extends to diagnostics for infectious diseases, pathogen detection, and neurological markers [16,17,18], culminating in point-of-care testing (POCT) devices [19,20].

The rapid evolution of these platforms has been accompanied by several highly comprehensive recent reviews. For instance, the recent literature has thoroughly explored the integration of polymer nanocomposites to enhance electrochemical signal transduction [21], polymer-mediated signal amplification mechanisms and structural optimization [22], and the surface functionalization of two-dimensional materials to improve biological compatibility [23]. While these recent contributions provide excellent foundations regarding material synthesis and outline general analytical figures of merit, they generally lack a deep, comparative analysis of how these devices perform when transitioned from idealized conditions into complex, real-world matrices.

To address this specific gap, this critical review provides an in-depth meta-trend analysis of polymer-based biosensors with a strict focus on analytical performance in real samples. Unlike existing recent reviews that primarily prioritize material synthesis, signal enhancement mechanisms, or broad qualitative overviews [21,22,23], this work critically scans all major important analytical parameters across a vast panorama of real samples. By rigorously evaluating the quantitative metrics and strictly analyzing the behavior of analytical parameters, this work determines the true readiness of these biosensors for real-world deployment.

### 1.1. Literature Organization and Meta-Data Processing

Biosensor transition from idealized buffer solutions to complex real-world matrices represents a critical challenge. Buffer studies establish theoretical limits of detection (LOD) but fail to account for matrix effects including biofouling, non-specific adsorption, and electrochemical interference.

**Figure 1:**
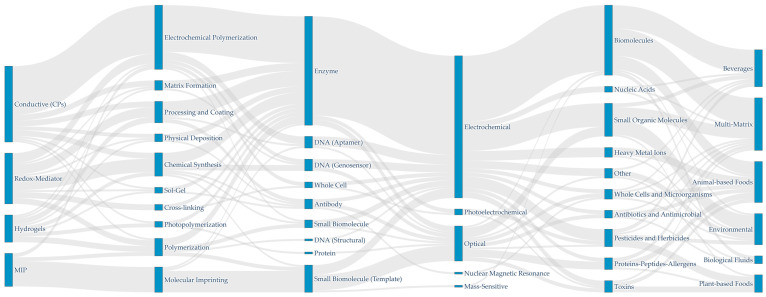
Macroscopic methodological flows in polymer-based biosensing. Sankey diagram illustrating the predominant technological pathways from polymer manufacturing techniques (left) to the integration of specific biorecognition elements, subsequent signal detection methods, and ultimate target molecule applications (right).

**Figure 2:**
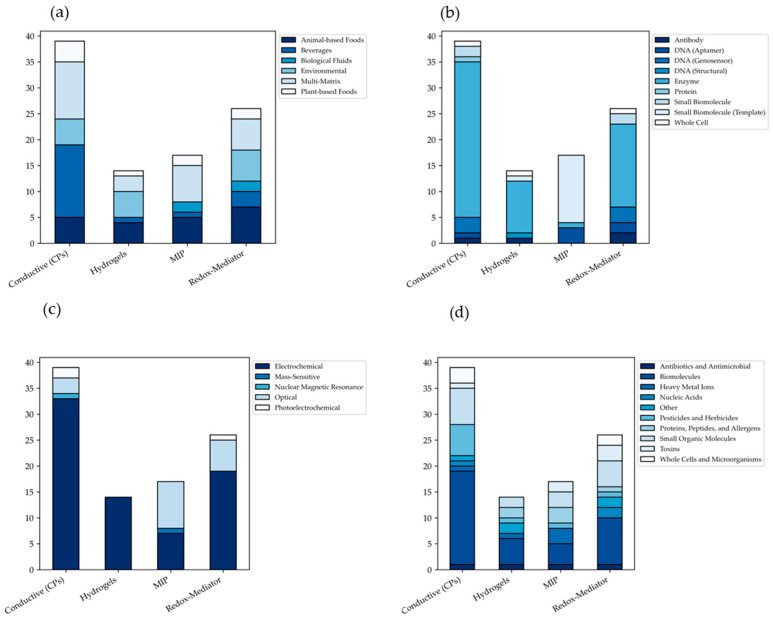
Analytical deployment and architectural trends of polymer-based biosensors. Multi-panel stacked bar charts detailing the functional distribution of the four primary polymer classes across different sensor design parameters: (**a**) target complex matrices, highlighting environmental applicability; (**b**) implemented biorecognition strategies, contrasting biological receptors with synthetic templates; (**c**) primary transduction mechanisms; and (**d**) categories of specific target molecules monitored by each polymer type.

To quantify how modern architectures overcome these hurdles, this review screened more than 1500 records from the Scopus database, selecting 96 original research articles published between 2015 and 2025. Extracted information was organized and processed using the Pandas library (version 2.3.3) in Python (Version 3.13) [24]. Methodological flows are visualized in a Sankey diagram (Figure 1) generated with Plotly (version 6.5.2) [25]. Cross-tabulated categorical data on biosensor applicability and transduction mechanisms appear in stacked bar charts (Figure 2) created with Matplotlib (version 3.10.8) and Seaborn (version 0.13.2) [26,27].

Figure 1 shows predominant methodological pathways. Electrochemical polymerization serves as the primary manufacturing route for conductive and electroactive polymers (CPs). This single-step fabrication method yields robust transducer surfaces and supports direct enzyme immobilization. Molecularly imprinted polymers follow a distinct pathway, relying on molecular imprinting techniques with biomolecule templates rather than biological receptors.

The reviewed literature can be divided into four primary material strategies. Each class represents a distinct approach to signal transduction and matrix management:

- Conductive and Electroactive Polymers (CPs) as Signal Transducers comprising the largest segment of the review [28,29,30,31,32,33,34,35,36,37,38,39,40,41,42,43,44,45,46,47,48,49,50,51,52,53,54,55,56,57,58,59,60,61,62,63,64,65,66]. These polymers facilitate the direct transduction of biological signals into electrical signals due to their inherent conductivity and π-conjugated structures. Key properties driving their widespread use include high electrical conductivity, excellent biocompatibility, the ability to be electrochemically polymerized, and a high surface area optimized for direct enzyme immobilization.
- Functional and Redox-Mediator Polymers represented by 26 studies [67,68,69,70,71,72,73,74,75,76,77,78,79,80,81,82,83,84,85,86,87,88,89,90,91,92]. These materials are specifically designed to shuttle electrons between the biological recognition element (such as an enzyme) and the electrode. They can also provide specific chemical functionality, like ion-exclusion or pH sensitivity. Their effectiveness relies on the presence of redox-active centers (e.g., Osmium or Ferrocene complexes), charge-carrying groups, and excellent film-forming stability.
- Hydrogels and Stimuli-Responsive Immobilization Matrices were exemplified by 14 studies [93,94,95,96,97,98,99,100,101,102,103,104,105,106]. These three-dimensional networked polymers can absorb large amounts of water to provide a biomimetic, protective environment for protein entrapment. Their analytical utility stems from their high-water content, tunable porosity, excellent biocompatibility, and responsiveness to external stimuli such as light, pH, or temperature.
- Molecularly Imprinted Polymers (MIPs) as Synthetic Recognition Elements encompassing 17 studies [107,108,109,110,111,112,113,114,115,116,117,118,119,120,121,122,123], this category focuses on “synthetic antibodies.” These are created by polymerizing monomers in the presence of a target template molecule, leaving behind customized binding cavities once the template is removed. MIPs offer high lock-and-key selectivity, chemical stability, thermal robustness, and cost-effectiveness compared to fragile biological receptors.

Selection among these classes directly dictates transduction pathway. Electrochemical polymerization serves as the primary manufacturing route for conductive polymers. Poly(3,4-ethylenedioxythiophene) (PEDOT) and polyaniline (PANI) are favored for lowering oxidation potentials and minimizing interference from co-existing species.

For targets where biological enzymes are unstable or unavailable, MIPs provide robust alternatives. While CPs deploy predominantly with enzymatic receptors for monitoring biomolecules across beverages and multi-matrix scenarios, MIPs target heavy metal ions and toxins in environments where biological receptors degrade rapidly.

Table 1 summarizes quantitative metrics and target applications for direct comparison of polymer architecture performance in complex environments.

Architectural design determines functional success across testing environments. Figure 2 provides comprehensive breakdown of these trends through multi-panel stacked bar analysis.

- Matrix Applicability (Figure 2a): The deployment of sensor types is highly dependent on the target environment. CPs demonstrate vast applicability, being predominantly deployed in beverage analysis [33,34,35,36,37,38,39,40,41,42,43,44,45,46] and broad multi-matrix scenarios [52,53,54,55,56,57,58,59,60,61,62]. In contrast, MIPs display a resilient presence across highly complex, fouling-prone matrices, including significant utilization in animal-based foods [107,108,109,110,111] and multi-matrix environments [115,116,117,118,119,120,121]. Hydrogels are more evenly distributed, showing strong utility in environmental [98,99,100,101,102] and animal-based food applications [93,94,95,96].
- Biorecognition Strategies (Figure 2b): This panel highlights the overwhelming reliance on enzymatic biorecognition within CP frameworks. Thirty studies employ this specific pairing [28,31,32,35,36,37,38,39,40,41,42,43,45,46,47,49,51,52,53,54,55,56,57,59,60,61,62,63,64,66], driven by the synergistic ability of CPs to facilitate rapid electron transfer from redox enzymes. Similarly, hydrogels rely heavily on enzymes (9 studies) [93,95,96,97,98,101,103,104,105]. Alternatively, MIPs largely bypass biological fragility, relying primarily on biomolecule templates (13 studies) to create synthetic recognition cavities [108,110,112,113,114,115,116,117,118,119,120,121,123], though they also successfully integrate DNA aptamers [107,109,111] or enzymes [122] for specific targets.
- Transduction Mechanisms (Figure 2c): When examining signal detection, electrochemical methods remain paramount across the entire field. CPs [28,30,31,32,33,34,35,36,37,38,39,40,41,42,43,44,45,46,47,49,51,53,54,55,56,57,59,60,61,62,63,64,66], Hydrogels [93,94,95,96,97,98,99,100,101,102,103,104,105,106], and Redox-Mediator polymers [67,69,70,73,74,76,77,79,80,81,82,83,85,86,87,88,89,90,92] heavily favor electrochemical pathways due to their inherent cost-effectiveness and capacity to perform sensitive measurements in turbid samples. Optical methods carve out highly specialized niches, serving as a significant secondary detection method for MIPs [110,112,114,115,116,117,121,122,123] and specific redox platforms [71,72,84,91].
- Target Molecules (Figure 2d): Analyzing the specific targets uncovers a clear bifurcation in research priorities. CPs are primarily designed for the continuous or rapid monitoring of abundant biomolecules [28,30,32,38,39,40,41,42,43,45,46,51,54,56,57,59,60,62] and small organic molecules [33,36,47,49,50,53,65]. Conversely, MIPs are strategically utilized for the highly selective, trace-level detection of severe contaminants, prominently targeting heavy metal ions [107,109,111], specific proteins [110,115,121], and toxins [108,123].

### 1.2. Analytical Realities: Metrics vs. Matrix

The meta-data demonstrate that while electrochemical detection dominates across all polymer types, extreme sensitivity frequently introduces practical trade-offs. Sensor utility depends on how polymer architecture manages the transition from buffer solutions to real-world matrices.

CP-based electrochemical biosensors achieve LODs from sub-micromolar to ultra-trace levels with rapid response times suitable for high-throughput screening of abundant biomolecules [28,30,32]. However, precision remains moderate; while highly variable across matrices (0.1% to 20%), RSD is typically below 6.5%. Bare conductive films with direct enzyme immobilization often show short to moderate shelf-lives due to bioreceptor denaturation in lipid-rich or protein-rich matrices.

Redox-mediator polymers demonstrate versatility through optical [68,71,72] and electrochemical [67,69,70] transduction. They deliver excellent accuracy and high selectivity for pathogenic whole cells [71], toxins [67], and nucleic acids [70]. Trade-offs include delayed operational response times [69,71] and limited short-term stability when relying on delicate genetic material or complex enzyme architectures [70].

Hydrogels actively combat biofouling in protein-heavy samples such as milk or serum. Their biocompatible hydrophilic microenvironments preserve enzyme kinetics and prevent rapid surface passivation. Ultra-wide dynamic measurement ranges and exceptional analytical precision with RSDs routinely below 5% [93,94,95,96] make them suitable for stable detection of antibiotics [93], allergenic proteins [94], and essential biomolecules [95,96] in complex fluidic foods.

MIPs demonstrate clear superiority when applications demand absolute stability and specificity over prolonged periods. In highly fouling matrices including animal foods and wastewater, these synthetic receptors report excellent recovery rates for trace-level contaminants [107,108,109,111]. Specificity derives from rigid molecular cavities rather than fragile protein folding. High precision (typically below 6% RSD) enables the detection of heavy metal ions [107,109,111] and toxins [108,123]. The primary limitation is a delayed operational response resulting from slow analyte diffusion through dense synthetic networks [107].

These findings indicate that technological readiness depends on a polymer’s ability to filter physical interferences, resist biological fouling, and maintain a reliable dynamic range during actual sample analysis, rather than merely achieving the lowest LOD in an ideal buffer.

To enable direct comparison across heterogeneous studies, quantitative metrics were harmonized by standardizing concentration measurements to a uniform micromolar scale and temporal data to seconds. Additionally, a comprehensive classification framework was developed to group sensors into distinct performance tiers based on critical parameters like detection limits, dynamic range, reliability, and operational speed. Data integrity measures were applied throughout the process to correct chemical outliers, consolidate redundant metrics, and transparently document missing information.

## 2. Polymer Functionality, Fabrication, and Detection Methodologies

### 2.1. Functional Classification of Polymers in Biosensing

Polymers have evolved from passive immobilization supports into active functional elements that dictate analytical capability. Literature from 2015 to 2025 categorizes these materials into three distinct functional roles: signal transducers, redox mediators, and synthetic recognition elements.

**Figure 3:**
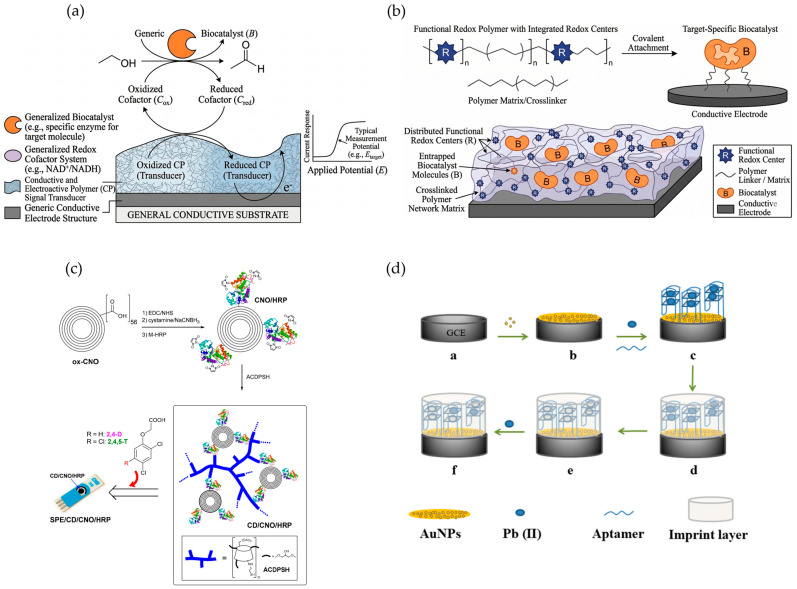
Design, assembly, and mechanisms of advanced multifunctional polymer biosensors. (**a**) Generalized signal transduction pathway where a Conductive and Electroactive Polymer (CP) relays electrons from an enzymatic redox cycle to an electrode. (**b**) Molecular components and 3D cross-section of a functional polymer matrix featuring entrapped biocatalysts and distributed redox centers. (**c**) Hierarchical nanocomposite structure integrating a branched polymer (ACDPSH), carbon nano-onions (CNOs), and HRP enzymes. Adapted from Sok and Fragoso [102]. (**d**) Fabrication sequence of an imprinted electrochemical aptasensor for Pb(II) detection, showing template assembly, polymerization, and removal for specific rebinding. Adapted from Zhu et al. [109].

As reported in Figure 3a, conductive polymers lower detection overpotential, filtering electrochemical noise from complex matrices. PEDOT and PANI operate as dominant transducers, catalyzing oxidation of intermediate molecules like NADH or H_2_O_2_ at low potentials (approximately 0.1 V versus Ag/AgCl) [40,45]. Low-potential operation minimizes interference from electroactive compounds such as ascorbic and uric acids [54] or phenolic compounds like catechol [47]. Without structural reinforcement using carbon nanotubes or graphene, pure films suffer from slow diffusion rates and mechanical instability [42,43].

As shown by Figure 3b, functional redox polymers facilitate electron transfer between enzyme active sites and electrode surfaces. Electron hopping renders oxidases independent of ambient oxygen fluctuations, enabling accurate quantitative analysis in biological fluids with variable oxygen concentrations such as milk or blood serum [69,87,90].

MIPs and stimuli-responsive immobilization matrices ensure signal fidelity (Figure 3c,d). To optimize performance, enzymatic systems often utilize hierarchical nano-composite materials, such as a CD/CNO/HRP assembly [102]. Chitosan and functionalized hydrogels create hydrophilic exclusion layers that prevent biofouling by ambient proteins, though protective mechanisms introduce diffusion barriers that can dampen sensitivity [100,111]. While protective layers help preserve enzymatic sensors, MIPs inherently offer superior structural stability in harsh environments where biological receptors otherwise denature rapidly. Recent innovations have transitioned MIPs from bulk polymers to surface-imprinted layers on optical fibers for food safety monitoring [115,117]. However, while MIP cavities provide exceptional selectivity [119], they exhibit slower binding kinetics than their enzymatic counterparts, requiring longer sample incubation times.

### 2.2. Manufacturing Strategies and Sensor Reproducibility

Manufacturing strategy determines film thickness, porosity, spatial resolution, and ultimately sensor reproducibility measured by RSD and operational shelf-life.

Electrochemical polymerization remains the dominant fabrication strategy, utilized in over 35% of the reviewed works, particularly for conductive polymers like PANI and PEDOT [32,45]. The critical advantage of this technique is its self-limiting nature. By precisely controlling the charge passed through coulometry or adjusting the number of cyclic voltammetry scans, researchers can tune the polymer film thickness with nanometer precision [39]. Localized direct deposition allows simultaneous entrapment of enzymes within three-dimensional conductive matrices, electrically integrating enzyme active centers while shielding them from proteases [41,49]. Overly dense films that hinder substrate diffusion remain a limitation. Successful implementations like PANI-PAAMPSA networks for ethanol detection rely on specific dopants that maintain porosity for rapid response times under five seconds without compromising electrocatalytic NADH oxidation at low potentials [45].

Conversely, solution processing techniques, including drop-casting and photopolymerization, offer massive scalability but struggle with surface homogeneity due to inhomogeneous drying patterns. To mitigate these drying defects, composite inks incorporating cyclodextrins are routinely employed to stabilize the suspension and uniformly increase the electroactive surface area [102]. While solution processing heavily facilitates the mass production of screen-printed electrodes [70], it generally yields higher RSDs, typically exceeding 5%, compared to the strict spatial control afforded by electropolymerization.

In the development of MIPs, the manufacturing focus has firmly shifted away from traditional bulk polymerization [120], which historically suffers from slow mass transfer and deeply embedded target sites, toward advanced surface imprinting, electrodeposition, and core–shell architectures. For instance, the electropolymerization of monomers like resorcinol [108] or dopamine [107] via cyclic voltammetry allows for precise film thickness control directly on the transducer surface. Additionally, the self-polymerization of dopamine in weakly alkaline media is now widely used to create conformal imprinted layers on magnetic iron oxide nanoparticles [110] or microplates [122]. Other sophisticated, highly tailored manufacturing strategies include sol–gel co-condensation using silane precursors (APTES and TEOS) [112], rapid photopolymerization under UV light [113], and free-radical core–shell imprinting around magnetic nanoparticles [114,118] or optical fibers [115].

Despite these advancements, the persistent challenge in this domain remains the template removal process. Incomplete template extraction results in high background signals, whereas overly aggressive washing protocols can physically collapse the fragile imprinted cavities.

To contextualize how these manufacturing methods pair with specific recognition elements in real-world scenarios, Table 2 summarizes the dominant analytical configurations deployed in complex food matrices.

### 2.3. Biorecognition Elements and Polymer Synergy

Biorecognition element selection determines required polymer physicochemical architecture. Enzymes dominate the field, utilized in 59% of studies, because catalytic turnover provides massive signal amplification critical for detecting trace analytes without sample pre-concentration.

For oxidases including glucose oxidase or xanthine oxidase, polymer functions as an electrical conduit. Conductive polymers like PEDOT, poly(SBTz) and PANI pair with these enzymes to facilitate direct electron transfer at low overpotentials, bypassing interference from ascorbic acid in fruit juices and wines [40,42,46].

For dehydrogenase-based sensors, the polymer must act as a localized reservoir for cofactor regeneration. Redox-active polymers or engineered composites electrocatalytically oxidize NADH at potentials as low as 0.1 V, preventing electrode fouling in heavy matrices like grape must [45,74].

Researchers have repurposed enzyme inhibition by aggressive matrix components for environmental monitoring. Targeted inhibition of acetylcholinesterase within polymer scaffolds detects pesticides and heavy metals [55,64]. The polymer’s role is protective, creating hydrophilic microenvironments that preserve baseline enzyme activity while allowing target inhibitor diffusion.

When long-term biological stability limits applications, synthetic receptors and nucleic acids emerge as reliable alternatives. MIPs are favored for targets with no natural enzyme or in extreme environments where proteins denature instantly. Recent literature demonstrates MIPs successfully enrich pesticides from vegetable matrices, offering synthetic specificity rivaling natural biological affinity [122].

For macromolecular targets like whole bacterial cells or complex food allergens where direct electron transfer is impossible, aptamers and antibodies are utilized [58,71]. Cationic polymers like poly(diallyldimethylammonium chloride) (PDDA) and poly-L-lysine serve as high-density electrostatic anchors for proper receptor orientation and maximum spatial capture efficiency [68,71].

Enzymatic systems offer catalytic sensitivity and integrate with conductive polymers for direct electrochemical readout but remain limited by short shelf-lives [55,73]. Synthetic receptors and aptamers solve the stability crisis and enable long-term device regeneration but lack intrinsic signal amplification, often requiring complex secondary transduction schemes for comparable LODs.

### 2.4. Detection Methodologies in Complex Matrices

Electrochemical methods dominate the analytical landscape, representing 79% of deployed systems. This dominance stems from inherent physical compatibility with turbid, colored matrices like whole blood [54,86] or fruit pulps [34,35] where standard optical paths are obstructed. The primary analytical challenge in opaque matrices is distinguishing target signal from background noise generated by ambient electroactive interferents. Modern designs leverage the electrocatalytic properties of functional polymers to shift required detection windows to lower potentials, thermodynamically excluding environmental interference [45,74,86].

Most successful electrochemical strategies employ complex composite architectures combining PEDOT or PANI with carbon nanotubes [40,42,47]. This pairing ensures rapid electron transfer while maintaining hydrophilic external interfaces that resist immediate protein fouling. Amperometry remains the standard for quantitative analysis due to rapid data acquisition.

Electrochemical impedance spectroscopy (EIS) has emerged as a powerful label-free alternative for detecting biological targets, operating at an applied potential of 0.0 V to eliminate background redox noise [95]. Despite advanced polymer engineering, a persistent limitation across the field is the reliance on sample dilution protocols. Most operational sensors still require dilution to prevent irreversible surface passivation [35,104].

Optical biosensors represent a secondary niche, comprising 17% of reviewed platforms, designed for rapid instrument-free screening. Engineered polymers like polydiacetylene induce visible colorimetric transitions upon target binding, offering immediate safety checks for pathogens in food processing environments without external power [29]. Reliability of optical methods is frequently compromised by the intrinsic pigmentation of matrices or biological fluids, requiring rigorous background data correction [39].

Emerging methodologies such as photoelectrochemical sensing [48,75] and nuclear magnetic resonance (NMR) detection [58] offer exceptional analytical specificity. In the case of NMR detection, the biosensor measures the spin-spin relaxation time (T_2_) of water protons. When target bacteria (such as *E. coli* O157:H7) bind to antibody-functionalized magnetic polyaniline nanoparticles, they form magnetic clusters. This clustering phenomenon accelerates the resonance signal decay of the surrounding water molecules, providing a highly sensitive readout that is largely unaffected by the optical transparency of the matrix [58]. However, despite their high specificity, these advanced techniques currently lack the field-deployability and cost-effectiveness of screen-printed electrochemical polymer arrays.

Detection methodology choice is dictated by target matrix physical properties: electrochemistry for accurate quantification in opaque fluids, optical platforms for qualitative screening in clear or processed media.

### 2.5. The Quantification and Reproducibility Deficit: A Meta-Analytical Critique

To explicitly differentiate our findings from purely descriptive reviews, this critical review driven by meta-analysis scrutinized the exact reporting of analytical parameters, revealing a stark reality. The prevailing literature prioritizes the pursuit of ultra-low Limits of Detection (LOD) at the severe expense of actual quantification, reproducibility, and real-world stability.

The most glaring deficit exposed by our analysis is the near-total absence of the Limit of Quantification (LOQ). Across the 96 evaluated studies, a staggering 91% fail to report LOQ. This omission is across all material classes missing in the vast majority of Conductive Polymer studies [29,30,31,32,33,34,35,36,37,39,40,41,42,43,45,46,48,49,50,51,52,53,54,55,56,57,58,59,60,61,62,63,64,65,66], Redox-Mediator platforms [67,68,69,70,71,73,74,75,76,77,78,79,80,81,83,84,85,87,88,89,90,91], and MIPs [107,108,109,111,112,113,114,115,116,117,118,119,121,123].

The consequences of this deficit are severe. LOD merely represents the statistical threshold at which a signal can be distinguished from background noise; it does not indicate the concentration at which an analyte can be reliably measured. By reporting trace and ultra-trace LODs while omitting LOQ, the field is effectively generating binary “yes/no” detectors rather than true quantitative biosensors. This fundamentally misrepresents technological readiness and explains why sensors that appear highly sensitive on paper routinely fail independent commercial validation where precise quantification is required.

Furthermore, 33% of the surveyed literature omits reproducibility and 30% precision relying on purely qualitative descriptions. Crucial precision metrics (such as inter-electrode and intra-electrode Relative Standard Deviation, RSD) are also partially absent in major portions of the Conductive [31,33,34,36,50,56,57,58,59,65], Redox [67,69,71,72,78,79,80,91], and MIP [107,108,109,110,112,114,115,117,118,123] literature.

When reproducibility is reported, a concerning number of platforms exhibit low precision, with RSDs exceeding 10% [28,77,90,121,122]. What this means for the field is brutal but necessary to acknowledge: we currently possess thousands of published “sensors” that are essentially irreproducible academic exercises. Without standardized reporting of manufacturing variance and inter-batch reliability, even the most innovative polymer synergy cannot transition from a benchtop prototype to a scalable technology.

This validation deficit extends to operational robustness. Despite targeting complex food and environmental matrices, long-term stability data is frequently either omitted or reveals ultra-short shelf-lives [28,36,39,46,62,76,78,114,115,123]. Similarly, response times are routinely left unreported across all polymer classes (e.g., Refs. [28,33,34,35,94,95,96,108,120]), blinding future researchers to the actual kinetic limitations of the proposed architectures.

Ultimately, a critical bottleneck in polymer biosensor research was found. The field has mastered the chemistry required to detect biomolecules at picomolar levels, but it has neglected the metrology required to make those measurements reliable.

## 3. Polymer Architectures for Food Matrix Analysis

### 3.1. The Matrix Challenge in Food and Beverage Analysis

The transition from idealized buffers to complex food matrices introduces barriers that compromise sensor performance. Polymer architectures must shift from simple immobilization supports to active physical filters, signal amplifiers, and steric shields. Methodological divergence is primarily based on the sample’s biological origin: animal-derived matrices present severe macromolecular fouling challenges, while plant-based matrices are limited by native electroactive interference.

Being inherently liquid, homogeneous, and often pH-buffered, they bypass the destructive homogenization steps required for solid tissues. However, beverages represent chemically aggressive environments with high concentrations of electroactive interferents, including ascorbic acid and polyphenols, alongside extreme pH levels. Consequently, the polymer role evolves from a passive mechanical binder to an active electrochemical filter. Literature from 2015 to 2025 establishes that Conductive Polymers (CPs) and redox polymers address thermodynamic interference by physically lowering required detection potentials. Acid-doped PANI and osmium-complexed redox wires facilitate the oxidation of intermediate molecules like NADH at potentials as low as +0.1 V. This low-voltage strategy effectively blinds biosensors to high-potential native antioxidants in wine and beer, enabling the unhindered monitoring of primary metabolites such as ethanol and glucose.

### 3.2. Animal-Based Food Matrices

In meat, seafood, and dairy, the primary analytical barrier is rapid electrode passivation by lipids and high-molecular-weight proteins. For quantifying spoilage markers in solid tissues, polymer selection is driven by the necessity to lower oxidation potentials and avoid background noise. Detecting xanthine in decomposing salmon utilizes organic electrochemical transistors (OECTs) based on p-type conducting polymers like p(g42T-TT) [28]. These architectures provide intrinsic signal amplification to overcome matrix noise, achieving a dynamic range of 5 to 98 µM. Standard amperometric composite sensors offer alternatives by creating anionic repulsion layers, such as electropolymerized Poly(L-aspartic acid) nanocomposites, which permit analyte diffusion while blocking negatively charged ambient proteins [73].

Dairy matrices present specific complications from high casein micelle and fat globule concentrations. Redox-active polymers, particularly osmium-complexed networks, facilitate highly efficient electron transfer for the direct detection of galactose [69]. Detecting trace contaminants like benzalkonium chloride or specific allergens in milk requires highly hydrophilic networks, such as photocrosslinkable gels or chitosan-nanocomposites, to create hydrated exclusion zones that repel non-specific protein adsorption [93,94].

For trace analytes where enzymatic recognition is unviable or easily inhibited, Molecularly Imprinted Polymers (MIPs) serve as robust synthetic alternatives. Chitosan-based MIPs actively lock the spatial conformation of aptamer-target complexes to detect heavy metal ions like lead and mercury in seafood [109,111]. Similarly, rigid porous organic polymers and polyresorcinol networks maintain cavity integrity against milk swelling pressure to detect hydrophobic toxins like Aflatoxin M1 at femtomolar concentrations [67,108]. Despite achieving high recovery accuracy (95–106%), MIPs struggle with bulk protein adsorption compared to hydrophilic hydrogels, indicating that true, direct analysis without sample pretreatment remains rare [110,111].

### 3.3. Plant-Based Food Matrices

Plant-based matrices (fruit pulp, tea, tubers) introduce distinct chemical hurdles. Unlike animal serum, plant tissues contain high concentrations of polyphenols, pigments, and organic acids that oxidize at standard sensing potentials. Therefore, the architectural strategy shifts heavily toward electrochemical filtering and optical evasion.

To bypass high background currents from native antioxidants in matrices like potato chips, optical transduction is frequently utilized. Cationic conjugated polymers, such as poly(fluorene-phenylene) (PFP), function as fluorescence resonance energy transfer (FRET) donors in DNA-based assays to detect non-electroactive targets like acrylamide [65]. This optical evasion achieves detection limits 0.16 μM (standard conditions) and 1.3 μM (in real samples) without the electrochemical noise common in starchy samples. Electrochemiluminescence (ECL) using polymer nanoparticles enables highly sensitive organophosphorus pesticide detection by measuring light emission, effectively isolating the signal from the electroactive background [66]. Colorimetric approaches measuring chromatic shifts upon target binding offer reliable screening for Aflatoxin B1 in corn and wheat extracts [91].

In strictly electrochemical applications, polymer physical morphology must be modified to filter physical debris. While standard conductive composites like PEDOT/MWCNT can lower oxidation potentials [64], hierarchical structures like phloroglucinol-based microporous organic polymers (OH-POFs) provide superior physical filtration. Their dense porosity protects electrode surfaces and stabilizes acetylcholinesterase, yielding femtomolar sensitivity for pesticide detection in fibrous vegetable extracts [63]. However, highly complex starchy matrices still force most sensors to rely on significant upstream buffer dilution and organic solvent extraction [92,123].

### 3.4. Architectural Selection Dictated by Beverage Matrix Hazards

The specific formulation of a beverage dictates the necessary polymer architecture, revealing distinct engineering pathways for high-sugar juices versus complex fermented wines.

In highly acidic and sugar-dense environments, hydrogels are heavily employed as essential buffering interfaces. These hydrogel networks physically protect delicate enzymes from acidic denaturation while modulating the diffusion of the dense sugar matrix. A critical trade-off is consistently identified in the literature between operational sensitivity and structural stability. Electrospun nanofibers, specifically Poly(vinyl alcohol) (PVA) matrices, offer exceptional surface area, resulting in rapid reaction kinetics and high sensitivity for immediate testing [97]. However PVA is highly hydrophilic and gradually dissolves in aqueous media, severely limiting long-term use. Conversely, dense cross-linked networks, such as methacrylate-based hydrogels, offer exceptional acid resistance and long-term mechanical stability but suffer from significantly slower, diffusion-limited response times.

In fermented beverages, the primary electrochemical antagonists are high concentrations of tannins and polyphenols, which rapidly passivate electrode surfaces. For tracking metabolites like ethanol, the electrocatalytic properties of conductive polymers are sufficient. Acid-doped PANI (PANI-PAAMPSA) structures enable NADH oxidation at extremely low potentials (+0.1 V), bypassing most grape must interference [45]. Similarly, thiolated polymers like Poly(L-Cysteine) facilitate the precise orientation of alcohol dehydrogenase, protecting the electrode while lowering the required oxidation potential to +0.35 V [74].

However, for detecting trace targets like dopamine or tyramine, where abundant tannins completely inhibit natural enzymatic activity, the architectural strategy pivots away from biology entirely toward Molecularly Imprinted Polymers (MIPs). To survive the aggressive alcohol and acid content of wine without swelling, researchers utilize rigid silica-based MIPs. When coupled with optical transduction methods, such as Lossy Mode Resonance (LMR), these rigid cavities solve the specificity problem, achieving an Imprinting Factor of approximately 6. This architecture effectively decouples the highly specific chemical recognition from the surrounding background noise [112].

### 3.5. The Extraction Bottleneck and Direct Analysis Limitations

Technological readiness regarding absolute sensitivity is remarkably high. Signal amplification techniques, such as photoelectrochemical DNA scaffolds detecting malathion at 2 pg/mL [75] and SERS platforms detecting Sudan I [123], enable polymers to overcome baseline sensitivity limitations. However, analytical reliability remains heavily restricted by the physical realities of sample extraction. Current biosensor surfaces are engineered to nanoscale precision but still depend on macroscopic physical homogenization, sonication, and liquid dilution to manage fats and fibers. Next-generation polymer biosensing must integrate polymer-compatible physical extraction methods, such as microneedle arrays [118], to ensure stability in field environments.

The beverage sector represents the highest technological maturity level for polymer-based biosensors. Because these sensors avoid physical damage from solid tissue extraction, they routinely achieve industrial-grade analytical precision, with RSD frequently below 2% [42,46,74]. However, experimental protocols reveal a persistent limitation: true, undiluted direct analysis remains unrealized. Despite electrocatalytic advances from conductive polymers and MIP specificity, virtually all successful deployment protocols still mandate sample dilution, typically ranging from 1:10 to 1:100. Dilution is required to buffer extreme pH, reduce sugar viscosity, and physically mitigate rapid bio-passivation and electrode fouling caused by polyphenols and tannins. Polymer engineering has solved thermodynamic selectivity through advanced electrocatalysis and structural imprinting, but kinetic fouling remains an unresolved bottleneck. Until polymer surfaces physically resist passivation in raw, undiluted concentrates, commercial deployment will remain limited.

In Table 3 the summary of the important analytical performance metrics for polymer biosensors applied to food and beverage matrices.

## 4. Environmental, Clinical, and Multi-Matrix Applications

### 4.1. The Matrix Challenge: From Ultra-Trace to Multi-Matrix Environments

Environmental monitoring presents an analytical paradox not present in food and beverage sectors. While food analysis struggles with bulk macromolecular fouling and beverage analysis targets high-abundance metabolites, environmental sensing demands detection of ultra-trace contaminants in femtomolar to picomolar ranges. These micropollutants exist within vast dynamic volumes of water and soil containing chemically aggressive backgrounds of humic acids, variable pH, and heavy metal ions. Sample dilution to suppress background noise is non-viable because it pushes target analytes below regulatory LOD requirements. Successful polymer engineering must pivot from passive transduction to active in situ signal amplification or rigid selective exclusion.

Simultaneously, despite the commercial dominance of medical biosensors, academic literature reveals limited application of polymer-based sensors directly to raw clinical fluids. This scarcity stems from rapid non-specific adsorption of high-molecular-weight proteins including albumin and globulins, which passivate electrode surfaces within seconds. The ultimate test of polymer structural resilience is cross-matrix versatility: the ability of a single sensor architecture to operate accurately across different hostile environments without recalibration. A polymer engineered to resist blood serum protein corona may rapidly degrade when exposed to acidic fruit juices or tannin-rich wine. Addressing this multi-matrix challenge requires highly versatile polymer interfaces. Recent studies have deployed single biosensor platforms across drastically different matrices, testing human serum alongside fruit juices, milk, and urine [54], or analyzing both cancer cell culture media and dairy products [119]. These applications rely on advanced polymer chemistries such as electropolymerized purpald films [54] or polyaniline polymerized on amyloid fibrils [119] that simultaneously repel bulky proteins, resist pH fluctuations, and filter common electroactive interferents including ascorbic and uric acids.

### 4.2. Signal Amplification via Conductive Composites in Environmental Monitoring

For detecting trace phenolic toxins amidst electrochemical noise from dissolved organic matter, CPs, specifically PEDOT, serve as foundational transducers. PEDOT is favored for stable conductivity at neutral pH, enabling low overpotential operation that excludes high-potential background interferents [47].

Pure CP films have limited surface area restricting sensitivity. For trace endocrine disruptors like bisphenol A (BPA) and complex pesticides, researchers have shifted to nanostructured composites. Modern architectures leverage stacking interactions between polymer backbones and aromatic pollutants to pre-concentrate analytes at electrode surfaces. Metal–organic coordination polymers (MOCP) integrated with carboxylated graphene create high-surface-area scaffolds detecting hydroquinone at 1.70 µM with superior selectivity against structural isomers [81]. Conjugated polymer nanoparticles (CPNPs) act as a peroxidase-mimicking catalyst, achieving nanomolar sensitivity (0.005 µM) for hydroquinone by maximizing catalyst-polymer contact area [50].

For emerging biological contaminants like antibiotic resistance genes with femtomolar regulatory limits, polymers function exclusively as amplification scaffolds. Atom transfer radical polymerization (ATRP) growing ferrocene-grafted polymer chains directly on electrodes creates three-dimensional conductive wires, amplifying gene signals down to 0.06 fM [85].

### 4.3. Synthetic Recognition: The Environmental MIPs

Enzymatic systems demonstrate excellent sensitivity but remain vulnerable to irreversible inhibition by non-target heavy metals in industrial wastewater. The field is increasingly pivoting to MIPs for required femtomolar sensitivity and environmental resilience.

A decisive shift from purely organic MIPs to inorganic-organic hybrid architectures bridges sensitivity gaps. For bisphenol S (BPS) detection, traditional electrochemical limits were surpassed by coupling host-guest MIP recognition with surface-enhanced Raman scattering (SERS). Zeolitic imidazolate framework (ZIF-8) encapsulated with silver nanofibers creates plasmonic traps that physically constrain analytes within electromagnetic hotspots, achieving an LOD of 1.36 × 10^−14^ M [116]. Optical hybrids achieve unmatched sensitivity but sacrifice field portability compared to standard electrochemical MIPs.

For portable electrochemical monitoring in soil and river water, cyclodextrin-based polymers serve as robust scaffolds. Carbon nano-onions (CNOs) integrated within polymerized cyclodextrin matrices stabilize enzymes like horseradish peroxidase, acting as conductive cages that facilitate electron transfer while preventing enzyme leaching that typically plagues soil analysis [102].

### 4.4. Continuous Flow Monitoring and Hydrogel Networks

For continuous in-stream environmental monitoring, analytical priority shifts to preventing bioreagent leaching. Diffusional redox mediators are rapidly washed away in flow regimes, rendering photo-crosslinked redox hydrogels more effective [101]. The field has pivoted to reagentless designs using electrostatic layer-by-layer assemblies. Combining cationic poly(diallyldimethylammonium chloride) (PDDA) with anionic Nafion creates electrostatic cages that stabilize enzyme-nanocarbon complexes, facilitating direct electron transfer (DET) and enabling continuous BPA detection without soluble mediators [83].

To overcome the sensitivity limits of standard enzymatic hydrogels, researchers have introduced stimuli-responsive DNA hydrogels. A polyacrylamide/DNA copolymer designed for Hg^2+^ detection utilizes the target analyte to trigger a structural phase transition. Hg^2+^ initiates a hybridization chain reaction (HCR) that crosslinks the hydrogel, decoupling the signal from simple binding events and achieving a picomolar LOD of 0.042 pM [99]. DNA hydrogels offer exceptional sensitivity via conformational switching but are susceptible to nuclease degradation in non-sterile environmental samples.

### 4.5. Permselective and Redox Polymers in Biological Fluids

To combat matrix effects across diverse samples, researchers have moved from passive diffusion layers to active permselective and redox polymers.

Sulfonated tetrafluoroethylene-based copolymers (Nafion) combined with nanomaterials electrostatically repel anionic proteins while permitting the diffusion of small target metabolites like pyruvate [77]. Increasing polymer density to block fouling introduces diffusion barriers that dampen the analytical signal. Redox-active polymers such as Azure A-doped chitosan [86] or osmium-complexed mediators [90] act as low-potential molecular wires, facilitating target detection without requiring high overpotentials. Low-voltage operation minimizes background oxidation of endogenous interferents that plague high-voltage detection [54].

### 4.6. Hydrogels as Protective Shields

When the primary hurdle is surface passivation across multiple matrices, hydrogels represent a structural bridge. The fundamental engineering challenge is selective permeability: designing polymer networks capable of excluding bulk proteins while maintaining rapid diffusion for small analytes.

Literature indicates a shift from passive encapsulation toward functional coordination polymers and programmable DNA hydrogels. Osmium-complexed redox polymers [87] and metal–organic coordination polymers (MOCPs) [81] mediate electron transfer while acting as rigid steric barriers against fouling.

DNA hydrogels have emerged as programmable scaffolds. Hybridization chain reaction (HCR) creates three-dimensional DNA networks functioning as highly hydrated electrode interfaces [85]. Negative charge density repels non-specific protein adsorption while providing massive surface area for analyte capture. The permeability-response paradox remains: increasing cross-linking density blocks biofouling but introduces physical barriers that dampen peak currents and slow temporal response.

### 4.7. Synthetic Recognition: Hybrid MIP Architectures in Clinical Matrices

Transition to clinical and multi-matrix fluids often degrades natural enzymes, prompting MIP use as stable synthetic receptors. For highly complex clinical targets, pure organic MIPs often provide insufficient selectivity.

For hydrophobic targets like cholesterol existing in serum bound to lipoproteins, physical imprinting alone cannot distinguish target from structural analog cholestanol [113]. Resolution is achieved by coupling MIP sensors with chemometric algorithms (N-PLS). This hybrid approach combines substitutes physical selectivity with electrochemical resolution, demonstrating that for steroidal clinical targets, MIPs function best as selective sensors rather than absolute filters [113].

For tracking antibiotics directly in plasma, the strategy shifts from in situ detection to magnetic extraction. Novel core–shell architectures using magnetic fluorescent MIPs allow analyte extraction from plasma prior to optical readout [114]. This separation successfully bypasses multi-matrix fouling but sacrifices continuous real-time monitoring capability.

### 4.8. Analytical Assessment and the Deployment Gap

Polymer chemistry for environmental sensing has reached remarkable maturity regarding absolute sensitivity, evidenced by the attomolar detection capabilities of advanced nanocomposites [85] and robust process control in automated flow systems [101]. However, critical review exposes a significant deployment gap. Most advanced studies rely on spiked tap water for validation [48,49,81]. While these tests yield excellent recovery rates (96–101%), they fail to account for irreversible biological and chemical inhibition caused by real wastewater effluents or bioactive sludge. Current technologies including smartphone-integrated polymer dots [80] and plant-penetrating microneedle arrays [118] excel as sensitive portable spot-check tools with operational resilience for autonomous long-term environmental monitoring.

Analysis of multi-matrix samples and biological fluids presents a similar paradox: exceptional quantitative accuracy with recovery rates of 94–116% coexists with technological underdevelopment for direct unassisted clinical application. The high accuracy reported in most studies is frequently an artifact of sample dilution. Samples are heavily diluted prior to testing to minimize fouling, or researchers rely on spiked synthetic fluids lacking the aggressive proteomic complexity of real patient samples. Bare conductive polymers cannot survive direct immersion in undiluted biological fluids.

The established path forward lies in structural innovation:

1. Microneedle-Integrated Systems: Bypassing standard flat electrodes, integrating MIPs onto microneedle arrays allows for non-destructive, in vivo monitoring, physically bypassing the extraction gap entirely [118].
2. Zwitterionic Anti-Fouling Skins: To enable direct analysis in whole blood or multi-matrix extracts, architectures must evolve to incorporate zwitterionic or highly hydrated hydrogel skins that provide a stealth effect against protein adsorption while maintaining necessary electron transfer kinetics, see Table 4.

Table 4 summarizes the main analytical findings of the environmental, clinical, and multi-Matrix Applications.

## 5. Critical Synthesis, Validation Gaps, and the Path to Commercialization

### 5.1. Technological Readiness and Validation Deficits

Critical synthesis of polymer biosensor literature from 2015 to 2025 exposes a dichotomy between material innovation and analytical validation. While polymer engineering has bridged sensitivity gaps routinely achieving femtomolar detection via signal amplification strategies, the field faces a quantification deficit. Meta-analysis reveals that while LODs are almost universally reported to demonstrate technological prowess, 91% of studies fail to report limits of quantification (LOQ).

This omission masks a critical reality: polymer sensors can detect analyte presence in complex matrices but often lack the signal-to-noise stability required for accurate quantification against a fluctuating background [48,50]. The validation deficit is compounded by a reproducibility and precision gap, frequently omitting sensor-to-sensor variance data. This correlates with manual fabrication methods. While electropolymerization offers precise spatial control for enzyme wiring [38,41], it is inherently difficult to scale without introducing batch-to-batch variance. Many reported devices represent non-replicable academic prototypes rather than viable commercial products.

### 5.2. Multi-Matrix Versatility

A significant deployment bottleneck is cross-matrix versatility. Polymer interface structural resilience determines the ability to operate accurately across different hostile environments without recalibration. A polymer engineered solely for blood serum may rapidly degrade in acidic fruit juices or tannin-rich fermented beverages.

Recent studies have attempted to deploy single biosensor platforms across drastically different matrices, testing human serum alongside fruit juices, milk, and urine [54], or analyzing both cancer cell culture media and dairy products [119]. These multi-matrix applications require advanced polymer chemistries that simultaneously repel bulky proteins, resist pH fluctuations, and filter common electroactive interferents. When physical polymers fail to provide universal selectivity, sensors must couple with chemometrics utilizing multi-way calibration algorithms to mathematically resolve overlapping signals from structural analogs [113].

To synthesize the strategies required to overcome these validation and matrix challenges, Table 5 outlines the primary analytical deficits and their corresponding architectural solutions.

### 5.3. Architectural Shifts for System Autonomy

To overcome the inherent resistivity of organic polymers and transition toward system autonomy, the recent literature emphasizes the construction of hierarchical nanocomposites. The integration of multi-walled carbon nanotubes and graphene with polymers like polyfluorene [36,57] or PEDOT [42] is no longer merely additive but synergistic. These hybrids create 3D conductive networks that lower electron transfer resistance and increase active surface area, solving the diffusion barrier problem by maintaining high conductivity while allowing analyte permeation [31,103].

Furthermore, the integration of chemometrics and smartphone readouts represents a massive leap toward decentralized diagnostics. While simple applications can quantify RGB values from colorimetric arrays [91], true innovation lies in coupling electrochemical data with machine learning [113]. The miniaturization of potentiostats into smartphone-controlled dongles bridges the gap between lab-quality voltammetry and field portability [70].

### 5.4. The Speed and Protection Trade-Off

The temporal resolution of a biosensor is governed by a fundamental physical contradiction: the polymer architectures required to shield electrodes from multi-matrix fouling invariably introduce diffusion barriers that retard signal acquisition. Response time is a direct consequence of polymer layer thickness.

Sensors achieving rapid responses typically minimize the diffusion path length by utilizing surface-confined conductive polymers or lateral flow formats, where kinetics are limited only by the sample flow rate rather than bulk diffusion [68,91]. Conversely, when polymers are engineered to filter complex interferents, speed is sacrificed for selectivity [96]. The most successful engineering workaround involves nanocapsulation. By wrapping individual enzyme molecules in a thin, cross-linked polymer shell rather than embedding them in a bulk hydrogel, researchers achieve robust protection against denaturing agents while maintaining rapid catalytic responses, proving that thin-film architectures are superior to bulk entrapment for dynamic monitoring [105].

### 5.5. Standardization Mandate and Future Outlook

The transition of polymer-based biosensors from academic curiosity to commercial viability hinges on a rigorous standardization mandate. The decade-long evolution signifies a decisive paradigm shift: polymers have graduated from passive immobilization matrices to active electrocatalytic filters capable of managing the matrix effect [36,69,87].

However, the field must completely deprecate theoretical LODs derived from idealized signal-to-noise ratios. Authors must report the LOQ in the target matrix alongside recovery rates [72,92,110]. Validation protocols must evolve from generic interference testing to matrix-specific stress panels, challenging blood sensors with globulins and food sensors with active proteases. Finally, the reliance on direct dilution methodologies must be replaced by integrated sampling hardware, such as microneedle arrays [118] and magnetic fluorescent cores [114], which physically bypass the extraction bottleneck.

## Figures and Tables

**Table 1:** Comparative analysis of four polymer classes detailing their dominant detection methods, biorecognition elements, dynamic range capabilities, sensitivity expressed as limit of detection (LOD), precision measured by relative standard deviation (RSD) with accuracy assessment, primary target applications, and corresponding references.

Polymer Class	Dominant Transduction (Count)	Primary Biorecognition (Count)	Primary Target Classes (Count)	Dynamic Range (Min–Max)	Average Precision (RSD)	Average Accuracy	Ref.
Conductive & Electroactive (CPs)	Electrochemical (33), Optical (3), Others (3)	Enzyme (30), DNA Genosensor (3), Others (6)	Biomolecules (18), Small Organics (7), Others (14)	Molecules: ~1 pM to 6 mM; Cells: 10^1^ to 10^8^ CFU/mL	0.1–20.0% (Typically < 6.5%)	92.0–145.7%	[28,29,30,31,32,33,34,35,36,37,38,39,40,41,42,43,44,45,46,47,48,49,50,51,52,53,54,55,56,57,58,59,60,61,62,63,64,65,66]
Functional & Redox-	Electrochemical (19), Optical (6), Others (1)	Enzyme (16), DNA Genosensor (3), Others (7)	Biomolecules (9), Small Organics (5), Others (12)	Molecules: 0.1 fM to 5 mM; Cells: 10^1^ to 10^7^ CFU/mL	0.20–9.28%	84.0–118.8%	[67,68,69,70,71,72,73,74,75,76,77,78,79,80,81,82,83,84,85,86,87,88,89,90,91,92]
Hydrogels & Responsive	Electrochemical (14)	Enzyme (10), Antibody (1), Others (3)	Biomolecules (5), Proteins/Peptides (2), Others (7)	Molecules: 0.1 pM to 540.6 µM; Mass: 0.01 pg/mL to 1 µg/mL	0.2–7.8%	71.0–120.0%	[93,94,95,96,97,98,99,100,101,102,103,104,105,106]
Molecularly Imprinted (MIPs)	Optical (9), Electrochemical (7), Others (1)	Template/Biomol. (13), DNA Aptamer (3), Others (1)	Biomolecules (4), Heavy Metals (3), Others (10)	Molecules: 0.1 pM to 200 µM; Mass: 0.5 ng/mL to 0.5 mg/mL	0.4–9.0%	79.7–119.6%	[107,108,109,110,111,112,113,114,115,116,117,118,119,120,121,122,123]

**Table 2:** Classification of polymer-based biosensors applied to food matrices showing the relationship between polymer class, biorecognition elements employed, manufacturing strategies utilized, detection methods implemented, specific target molecules analyzed, and corresponding references.

Polymer Class	Biorecognition Element	Manufacturing Strategy	Detection Method	Target Molecules	References
Conductive & Electroactive	Enzymes (Xanthine Oxidase, Glucose Oxidase), Structural Polydiacetylene (PDA)	Electropolymerization, Chemical Polymerization, Spin-coating, Photopolymerization	Electrochemical (Amperometry, DPV, CV), Optical	Xanthine, Cholesterol, Heavy Metals (via enzyme inhibition), Pathogens (*S. aureus*)	[28,29,30,31,32]
Functional & Redox-Mediator	Aptamers, Enzymes (Galactose Oxidase), DNA Probes, Antibodies	Chemical Synthesis, Crosslinking, Microwave-assisted	Electrochemical (Amperometry, CV, DPV), Optical	Aflatoxin M1, Antibiotics (Tetracyclines), Galactose, Pathogenic DNA/Cells, Allergens	[67,68,69,70,71,72]
Hydrogels & Responsive	Enzymes (Acetylcholinesterase, Choline Oxidase, Cellobiose Dehydrogenase), Antibodies	Photopolymerization, Drop-casting, Free Radical Polymerization	Electrochemical (Amperometry, SWV, EIS)	Biocides (BAC, DDAC), Food Allergens (β-lactoglobulin), Choline, Lactose	[93,94,95,96]
Molecularly Imprinted (MIPs)	Imprinted Cavities, DNA Aptamers (Hybrid MIPs)	Electropolymerization, Molecular Imprinting (Self-polymerization), Electrodeposition	Electrochemical (DPV, CV, EIS), Optical	Heavy Metals (Pb, Hg, Cd, As), Aflatoxin M1, Food Allergens (Tropomyosin)	[107,108,109,110,111]

**Table 3:** Analytical performance metrics for polymer biosensors applied to food and beverage matrices. Columns indicate specific matrix category, target molecules analyzed, biorecognition strategies employed, detection methods utilized, dynamic range expressed in micromolar (µM), colony forming units per milliliter (CFU/mL), or nanograms per milliliter (ng/mL), sensitivity expressed as limit of detection (LOD), and corresponding references.

Matrix Category	Main Target Molecules	Main Biorecognition Strategy	Main Detection Method	Dynamic Range	LOD	References
Animal-Based Foods	Xanthine, Cholesterol, Lactose, Choline, Pathogens, Heavy Metals	Enzymes, ssDNA, Aptamers, Structural Proteins	Electrochemical (OECT, DPV, Amperometry), Optical	3.0 × 10^−1^ to 6.5 × 10^6^ (µM or CFU/mL)	Sub-µM to 50 CFU/mL	[28,29,30,31,32,69,70,71,73,95,96]
Animal-Based Foods	Aflatoxin M1, Heavy Metals (Pb, Hg), Allergens (Tropomyosin), Biocides, Antibiotics	MIPs, Antibodies, Aptamers, Enzymes	Electrochemical (CV, EIS, DPV), Fluorescence	1.0 × 10^−5^ to 2.5 × 10^3^ (µM or ng/mL)	Femtomolar to trace ng/mL	[67,68,72,93,94,107,108,109,110,111]
Plant-Based Foods	Organophosphates, Malathion, Aflatoxin B1, Ochratoxin A, Sudan I	Enzymes (AChE), MIPs, Aptamers	DPV, ECL, Colorimetry, SERS	1.0 × 10^−9^ to 1.0 × 10^6^ (µM or ng/mL)	Ultra-trace to low µg/kg	[63,64,66,91,92,122,123]
Plant-Based Foods	Acrylamide, Ovalbumin	ssDNA, Antibodies	FRET (Optical), Voltammetry	6.7 × 10^−1^ to 1.6 × 10^1^ µM	Trace	[65,106]
Beverages	Ethanol	Alcohol Dehydrogenase/Oxidase	Amperometry	8.5 × 10^−3^ to 1.8 × 10^3^ µM	0.009 to 110 µM	[43,45,74,76]
Beverages	Glucose	Glucose Oxidase	Amperometry	0.5 to 3.0 × 10^3^ µM	0.7 to 41.0 µM	[39,40,41,46,97]
Beverages	Pathogens/DNA	Aptamers	EIS	10^2^ to 10^8^ CFU/mL	3 CFU/mL	[44]
Beverages	Trace Contaminants (Pesticides, Tyramine, Dopamine)	MIPs, Tyrosinase, AChE	Optical (LMR), Voltammetry (DPV)	3.0 × 10^−3^ to 1.0 × 10^2^ µM	Trace (0.027 µM for Dopamine)	[37,38,112]
Beverages	Pesticides	Aptamers	Photoelectrochemical	0.6 × 10^−2^ to 6.0 × 102 ng/mL	0.002 ng/mL	[75]

**Table 4:** Analytical performance metrics for polymer biosensors applied to environmental, clinical, and multi-matrix environments. Columns specify target molecules, biorecognition strategies, polymer architectures, detection methods, dynamic range expressed in micromolar (µM) or colony forming units per milliliter (CFU/mL), sensitivity as limit of detection (LOD), precision measured by relative standard deviation (RSD) with accuracy percentage, and corresponding references.

Matrix Category	Target Molecules	Biorecognition Strategy	Polymer Architecture	Detection Method	Dynamic Range	LOD	Precision/Accuracy	References
Environmental	Phenols & Catechol	Tyrosinase, Laccase	PEDOT, PA6/Pebax, MOCPs, PM1; Nafion	Amperometry, Voltammetry, Optical	1.0 × 10^−1^ to 4.0 × 10^2^ µM	Sub-µM (0.0007–11.0 µM)	High (1.5–6.7%)	[47,49,79,81,84]
Environmental	Endocrine Disruptors (BPA, BPS)	Tyrosinase, MIPs	ZIF-8/SERS hybrids, PDDA/Nafion	Amperometry, SERS (Optical)	2.8 × 10^−1^ to 4.5 × 10^1^ µM [83]	Femtomolar to 0.066 µM	High (2.2–5.1%)	[83,98,116]
Environmental	Herbicides & Pesticides	Cyanobacteria, Enzymes	P(SNS-Aniline), Cyclodextrin/CNOs	Photoelectrochemical, Amperometry	1.0 × 10^−1^ to 1.2 × 10^0^ µM	0.014 to 0.023 µM	Excellent	[48,102]
Environmental	Heavy Metals (Hg^2+^)	DNA Nanostructures	Polyacrylamide/DNA hydrogel	EIS	1.0 × 10^−7^ to 1.0 × 10^−2^ µM	0.042 pM	Moderate (4.1–7.8%)	[99]
Environmental	Bacterial Cells/Pathogens	Synthetic Boronic Acid	Carbonized Polymer Dots	Electrical Resistance	1.0 × 10^1^ to 1.0 × 10^7^ CFU/mL	6.3 CFU/mL	Data Not Available	[80]
Clinical/Multi-Matrix	Metabolites (Glucose, Pyruvate)	Oxidases (GOx, PyOx)	PIIDAnth, PFLO, poly(SNS-ERR), poly(BSeTT)	Amperometry, Voltammetry	1.0 × 10^1^ to 1.5 × 10^3^ µM	Sub-µM (0.012 to 81.0 µM)	High (0.1–4.9% RSD); 95–116% Accuracy	[56,57,59,60,77]
Clinical/Multi-Matrix	Multi-Matrix Phenols & Toxins (BPA, OA)	Tyrosinase, Aptamers	CPNPs, OPECT (PEDOT:PSS)	Amperometry, Photoelectrochemical	1.0 × 10^−4^ to 3.0 µM	Trace (1.25 × 10^−5^ to 0.017 µM)	High (4.2–10% RSD); 92–103% Accuracy	[52,53]
Clinical/Multi-Matrix	Multi-Matrix Biomarkers (AA, UA, L-Cys)	Non-enzymatic	Electropolymerized Purpald	Voltammetry (DPV)	3.3 × 10^0^ to 7.6 × 10^2^ µM	Trace (0.137 to 0.392 µM)	High (0.25% RSD); 95–105% Accuracy	[54]
Clinical/Multi-Matrix	Steroids & Lipids (Cholesterol)	MIPs + Chemometrics	MWCNT/AuNP/MIP nanocomposites	Voltammetry (DPV)	1.0 × 10^−2^ to 8.0 × 10^0^ µM	0.01 µM	High (2.8–3.0% RSD); 94–105% Accuracy	[113]
Clinical/Multi-Matrix	Antibiotics & Drugs (Doxycycline)	Fluorescent MIPs	Magnetic core–shell MIPs	Fluorescence Quenching	2.0 × 10^−1^ to 6.0 × 10^0^ µM	0.117 µM	Data Not Available; 88–107% Accuracy	[114]
Clinical/Multi-Matrix	Neurotransmitters (Glutamate, Tyramine)	Dehydrogenases, Tyrosinase	Azure A/Chitosan, PDDA/Nafion	Voltammetry, Amperometry	Up to 1.3 × 10^2^ µM	1.5 to 3.3 µM	Moderate; ~99.5% Accuracy	[86,89]

**Table 5:** Analysis of key analytical deficits limiting polymer biosensor commercialization with corresponding traditional limitations, advanced polymer solutions developed to address each challenge, required validation protocols for regulatory acceptance, and supporting references. Each row represents a distinct analytical challenge requiring specific methodological solutions for successful technology transfer from laboratory prototypes to commercial devices.

Analytical Challenge	Traditional Limitation	Advanced Polymer Solution	Validation Requirement	References
Quantification Deficit	Reporting theoretical LOD in ideal buffer solutions.	Integrated antifouling skins to stabilize baseline noise.	Mandatory reporting of LOQ in undiluted samples.	[72,92,110]
Multi-Matrix Problem	Sensor fails when moved from blood to acidic juice matrices.	Chemometric resolution (N-PLS) and zwitterionic universal filters.	Cross-matrix interference testing (pH, proteins, tannins).	[54,113,119]
Reproducibility Gap	Manual drop-casting causes high batch-to-batch variance.	Standardized electropolymerization and automated screen-printing.	Reporting Inter-Electrode RSD.	[38,74,86]
Permeability Paradox	Thick hydrogels block fouling but cause slow signal response.	Nanocapsulation of single enzymes in thin polymer networks.	Reporting response times alongside sensitivity metrics.	[96,105]
Leaching in Flow Systems	Diffusional mediators wash away during continuous monitoring.	Covalent enzyme wiring using transition metal coordination polymers.	Operational stability metrics (cycles until failure).	[87,88,90]

## Data Availability

No new data were created or analyzed in this study.

## Abbreviations

The following abbreviations are used in this manuscript:

ATRP	Atom transfer radical polymerization
AuNP	Gold nanoparticles
BPA	Bisphenol A
BPS	Bisphenol S
CNOs	Carbon nano-onions
CPNPs	Conjugated polymer nanoparticles
CPs	Conductive and Electroactive Polymers
CV	Cyclic Voltammetry
DET	Direct electron transfer
DPV	Differential Pulse Voltammetry
ECL	Electrochemiluminescence
EIS	Electrochemical Impedance Spectroscopy
FRET	Fluorescence resonance energy transfer
HCR	Hybridization chain reaction
LMR	Lossy Mode Resonance
LOD	Limit of detection
LOQ	Limit of quantification
MIP	Molecularly imprinted polymer
MOCP	Metal–organic coordination polymers
MWCNT	Multi-walled carbon nanotubes
NADH	Nicotinamide adenine dinucleotide
N-PLS	N-way partial least squares (chemometric algorithm)
OECT	Organic electrochemical transistors
OH-POFs	Phloroglucinol-based microporous organic polymers
OPECT	Organic photoelectrochemical transistors
PANI	Polyaniline
PDDA	Poly(diallyldimethylammonium chloride)
PEDOT	Poly(3,4-ethylenedioxythiophene)
POCT	Point-of-care testing
PVA	Poly(vinyl alcohol)
RSD	Relative standard deviation
SERS	Surface-enhanced Raman scattering
SWV	Square Wave Voltammetry
ZIF-8	Zeolitic imidazolate framework
